# A dual enhanced stochastic gradient descent method with dynamic momentum and step size adaptation for improved optimization performance

**DOI:** 10.1038/s41598-025-24689-y

**Published:** 2025-11-18

**Authors:** Mohamed A. Mokhtar, Mohamed Fathy, Yasser A. Dahab, Emad A. Sayed

**Affiliations:** 1 Basic and Applied Science Department, College of Engineering and Technology, Arab Academy for Science, Technology and Maritime Transport, Aswan, Egypt; 2 Basic and Applied Science Department, College of Engineering and Technology, Arab Academy for Science, Technology and Maritime Transport, Cairo, Egypt; 3 Computer Science Department, College of Computing and Information Technology, Arab Academy for Science, Technology and Maritime Transport, Aswan, Egypt; 4 Department of Physics& Engineering Mathematics, Faculty of Engineering-Mattaria, Helwan University, Cairo, Egypt

**Keywords:** Stochastic gradient descent, Adaptive momentum, Adaptive step size, Gradient-based optimization, Machine learning optimization, Applied mathematics, Computer science

## Abstract

In modern machine learning, optimization algorithms are crucial; they steer the training process by skillfully navigating through complex, high-dimensional loss landscapes. Among these, stochastic gradient descent with momentum (SGDM) is widely adopted for its ability to accelerate convergence in shallow regions. However, SGDM struggles in challenging optimization landscapes, where narrow, curved valleys can lead to oscillations and slow progress. This paper introduces dual enhanced SGD (DESGD), which addresses these limitations by dynamically adapting both momentum and step size on the same update rules of SGDM. In two optimization test functions, the Rosenbrock and Sum Square functions, the suggested optimizer typically performs better than SGDM and Adam. For example, it accomplishes comparable errors while achieving up to 81–95% fewer iterations and 66–91% less CPU time than SGDM and 67–78% fewer iterations with 62–70% quicker runtimes than Adam. On the MNIST dataset, the proposed optimizer achieved the highest accuracies and lowest test losses across the majority of batch sizes. Compared to SGDM, they consistently improved accuracy by about 1–2%**,** while performing on par with or slightly better than Adam in accuracy and error. Although SGDM remained the fastest per-step optimizer, our method’s computational cost is aligned with that of other adaptive optimizers like Adam. This marginal increase in per-iteration overhead is decisively justified by the substantial gains in model accuracy and reduction in training loss, demonstrating a favorable cost-to-performance ratio. The results demonstrate that DESGD is a promising practical optimizer to handle scenarios demanding stability in challenging landscapes.

## Introduction

Machine learning (ML) optimization is critical to the development of models that exhibit efficiency, scalability and superior performance. With the continuous advancement of modern ML approaches, the necessity of optimization in the training of complex models, such as deep neural networks, is becoming more essential. Recent developments, such as gradient-based approaches, adaptive learning rate strategies and stochastic optimization techniques, have greatly enhanced the performance of models in various applications like disease diagnosis^1–3^, photovoltaic power forecasting^4,5^, large language models training^6^ and speech recognition^7^. In addition, improving model performance plays a crucial role in enhancing computational efficiency, resulting in reduced training time, lower resource utilization and an increase in the accessibility and deployment of ML solutions in real life applications. Thus, the area of ML optimization remains a promising research area, offering significant ideas that enhance the progress of artificial intelligence across diverse sectors.

One of the powerful methods used in ML optimization is the gradient descent (GD), which minimizes the loss function $J(\theta )$ where $\theta$ are the model’s parameters by updating them in the negative direction of the gradient of the loss function ${\nabla }_{\theta }J(\theta )$. The step size to reach a local minimum is found by the learning rate $\alpha$.

1 $${\theta }_{i+1}=\theta -\alpha {\nabla }_{\theta }J\left(\theta \right).$$

The GD method has different variants according to the number of data samples that will be fed into the optimization process. Stochastic gradient descent (SGD) performs the parameters update using one data sample at a time instead of using all the data samples and completes one epoch i.e. one iteration, after finishing all the data samples.

2 $${\theta }_{i+1}=\theta -\alpha {\nabla }_{\theta }J\left(\theta ;{x}^{i};{y}^{i}\right),$$

where ${x}^{i}$ is a data sample and ${y}^{i}$ is its predictor.

At practical application of SGD, we can use $n$ data samples, not just one and hence we call it mini-batch gradient descent and $\text{n}$ is the batch size, but we still refer to it as SGD. SGD suffers slow convergence in highly non-convex loss functions where many local suboptimal points can be trapped in them. Dauphin^8^ discussed the problem of saddle points, i.e. points where one dimension slopes up and another slopes down. The gradient of a saddle point is close to zero in most dimensions, so SGD can be deceived that it reaches a good minimum point.

Stochastic gradient descent with momentum^9^ improves optimization by keeping a moving average of past gradients. This momentum term helps smooth out noisy or erratic updates, leading to more stable and efficient convergence. In practice, momentum reduces oscillations—particularly in regions with sharp curvature—while also speeding up progress in the right direction. Typically set to 0.9, the momentum factor controls how strongly past gradients influence the current step, striking a balance between speed and stability. Compared to plain SGD, SGDM is especially effective at navigating complex loss landscapes.

The following update rules governess SGDM where $\beta$ is the momentum factor. It adds a fraction of the update vector of the past time step ${\upsilon }_{i-1}$ to the current update vector ${\upsilon }_{i}$.

3 $${\upsilon }_{i}={\beta \upsilon }_{i-1}-\alpha {\nabla }_{\theta }J\left(\theta \right),$$

4 $${\theta }_{i+1}={\theta }_{i}+{\upsilon }_{i}.$$

In order to increase the pace of convergence of gradient descent, Yurii Nesterov developed the sophisticated optimization method known as Nesterov accelerated gradient (NAG)^10^. By addressing two significant drawbacks of conventional stochastic gradient descent (SGD), it improves upon it: slow convergence rates and susceptibility to unpredictable gradient behaviour in areas of high curvature (such as steep valleys or saddle points). NAG reduces excessive oscillations and overshooting by anticipating future gradients by computing updates at a predicted point, in contrast to traditional momentum-based approaches. This insight speeds up convergence to an ideal solution at a rate of $\mathcal{O}\left(1/{k}^{2}\right)$, which is substantially quicker than SGD’s $\mathcal{O}\left(1/k\right)$.

John Duchi created the adaptive optimization method Adagrad^11^, which uses the accumulation of previous gradient magnitudes to modify the learning rate for each parameter separately. In particular, it scales the learning rate in an inverse proportion to the total squared gradients that have been encountered thus far. This promotes more balanced learning by enabling fewer updates for parameters with often big gradients and larger updates for those with smaller gradients. Adagrad’s cumulative sum of squared gradients, however, is a major disadvantage since it steadily increases, which eventually lowers the effective learning rate. Eventually, this may result in vanishing updates, which would slow down convergence and make it more difficult for the model to break out of less-than-ideal solutions.

Adadelta was created by Matthew D. Zeiler to address Adagrad’s falling learning rate issue^12^**,** In order to maintain a more controlled update, Adadelta employs an exponentially decaying moving average of squared gradients rather than continuously accumulating all prior squared gradients, which may cause the learning rate to drop too much. This technique prevents the learning rate from continuously decreasing by making sure that earlier gradients gradually lose their effect. Adadelta also has the important advantage of eliminating the need for a manually chosen starting learning rate. It alternatively determines step sizes dynamically by calculating the ratio of the root mean square (RMS) of the current gradients to the RMS of the previous parameter changes. This built-in adjustment helps the model learn more steadily and adapt to changes along the way.

Geoffrey Hinton’s RMSprop method^13^ provides a sophisticated solution to the issue of rapidly declining learning rates that Adagrad faces. It uses a moving average of squared gradients to dynamically modify the learning rate, much like Adadelta. But its use of an exponentially decreasing average is what makes it unique. This strategy makes sure that the influence of older gradients gradually diminishes, giving preference to more recent information, as opposed to continuously gathering all historical gradients. This mechanism strikes a vital balance between stability for convergence and adaptation to new input by successfully preventing the aggressive, monotonous reduction in learning rate. Scaling the learning rate by the square root of this smoothed average is the procedure used to update each parameter. Its broad use in deep learning is mostly due to this procedure, which also contributes to its strong performance in non-stationary situations. The decay rate, which determines the contribution of previous gradients to the moving average, is a key hyperparameter controlling this process. It is usually set at 0.9.

Adaptive Moment Estimation, or Adam^14^, was introduced by Diederik P. Kingma and is a potent optimization algorithm that combines ideas from adaptive learning rate approaches such as RMSprop and momentum-based approaches. During training, it keeps track of two important statistics for every parameter:

1. The first moment, which is the mean of the gradients, functions similarly to momentum in facilitating smooth updates and quickening convergence in predictable directions.
2. By monitoring the amplitude of previous gradients, the second moment (uncentered variance of gradients) adaptively scales the learning rate for every parameter, avoiding updates that are too big or small.

Both moments are computed using exponentially decaying moving averages, allowing Adam to adapt quickly while retaining useful historical information. To counteract bias introduced in the early stages of training—where moment estimates tend to be small—Adam applies bias correction terms, ensuring more stable updates, especially in the initial iterations.

In recent years, metaheuristic optimization algorithms have also garnered a lot of interest because of their robust global search capabilities and derivative-free nature. Traditional techniques like genetic algorithms^15^, Particle swarm optimization^16^, Ant colony optimization^17^, and Differential evolution^18^ have been extensively used in a variety of fields. These foundations have led to the introduction of many new metaheuristic techniques, including those based on animal behaviors such as the Fishing cat optimizer^19^, Draco lizard optimizer^20^, Bighorn sheep optimization algorithm^21^, and Frigatebird optimizer^22^, human social behaviors like Painting Training-Based Optimization^23^, mathematics-based concepts like logarithmic mean-based optimization^24^, physics-based concepts like the Gyro fireworks algorithm^25^ and other paradigms such as fungal growth optimizer^26^ which demonstrate promising performance on complex nonlinear problems. While our study focuses on gradient-based optimizers tailored for machine learning tasks, metaheuristic methods remain highly relevant in the broader optimization research landscape and provide complementary perspectives for solving challenging problems.

The usage of nonlinear conjugate gradient (NCG) as an adaptive momentum equation has been presented in many works. Wang and Ye^27^ propose a novel adaptive momentum for SGD inspired by the nonlinear conjugate gradient method, the Fletcher-Reeves method^28^, that automatically adjusts based on gradient history, thereby eliminating the need for manually tuning a momentum hyperparameter. This adaptive scheme enables the use of significantly larger learning rates, accelerates convergence, and enhances overall training efficiency. Empirical results show that it improves classification accuracy and reduces errors on CIFAR-10 and CIFAR-100 – and bolsters adversarial robustness. Theoretical analysis further demonstrates that, on quadratic functions, the method is equivalent to conjugate gradient methods, underscoring its solid convergence properties.

Hao et al.^29^ uses concepts from the nonlinear conjugate gradient framework to offer a quick training technique for deep neural networks that adaptively manages the learning rate and momentum. Specifically, they present the conjugate gradient with quadratic line-search (CGQ) method, which uses a quadratic line-search to find the ideal step size and adjusts the momentum parameter dynamically in the style of a conjugate gradient (using a method akin to the Polak–Ribiere method^30^). By removing the need for laborious human hyperparameter adjustment and allowing for higher learning rates, this architecture improves generalization and speeds up convergence on image classification tasks. Their method produces faster convergence and higher test accuracy than conventional local solvers, as shown by actual testing, and theoretical conclusions ensure convergence in severely convex circumstances.

In addition to SGDM, Kobayashi and Iiduka^31^ propose a new optimizer that combines Adam’s adaptive moment estimation with nonlinear conjugate gradient updates to improve stochastic optimization in deep learning. Their approach, called “Conjugate-Gradient-Based Adam,” incorporates multiple conjugate gradient formulations into the momentum update, leading to more reliable descent directions. The authors provide convergence analyses for strongly convex problems under standard assumptions and back up their theory with experiments on text and image classification tasks. The results show that the proposed optimizer trains deep neural networks in fewer epochs while delivering generalization performance on par with, or better than, existing adaptive optimization methods.

This paper introduces the dual enhanced stochastic gradient descent (DESGD) method, which advances beyond existing adaptive momentum approaches such as Wang and Ye^27^ in the following key aspects:

- Dual adaptive mechanism: Unlike prior works that adapt only momentum or learning rate, DESGD simultaneously adapts both momentum and step size within the SGDM framework, enabling more responsive and stable convergence in complex loss landscapes.
- Novel truncation schemes: We introduce two practical methods—clipping and reciprocal truncation—to bound the adaptive momentum factor within [0,1), ensuring stability without sacrificing adaptivity.
- Lightweight step size adaptation: By using a cosine-based alignment measure between consecutive gradients, DESGD avoids expensive line searches, making it suitable for stochastic and high-dimensional optimization.
- Theoretical underpinnings: We provide a convergence analysis showing that DESGD maintains bounded updates and achieves a sublinear regret bound under standard assumptions.
- Extensive validation: DESGD is evaluated on both classical optimization benchmarks and real-world machine learning tasks, demonstrating superior performance over SGDM and Adam in terms of iteration count, runtime, and generalization accuracy.

This paper’s remaining sections are arranged as follows: The technique, including the algorithm and theoretical foundations of DESGD, is described comprehensively in Sect. “Methodology”. Section “Theoretical foundations and convergence properties” covered the mathematical foundations of the convergence properties. Neural network and numerical test experiments are presented in Sect. “Experiments and results”. Results, comparisons, and computing efficiency are provided in Sect. “Discussion”. Limitations and future directions are discussed in Sect. “Conclusion”‘s conclusion.

## Methodology

The conjugate gradient (CG) method^32^ was originally developed to solve the problem of linear equations systems $Ax=b$ where *A* is the coefficient matrix, and this problem can be formulated to be a problem of convex quadratic function minimization defined by

5 $$f\left(x\right)=\frac{1}{2}{x}^{T}Ax-{b}^{T}x.$$

According to this concept, many extensions have been designed to solve general nonlinear functions under the name of nonlinear conjugate gradient (NCG) which has the form

6 $${\theta }_{i+1}={\theta }_{i}+{\alpha }_{i}{p}_{i},$$

7 $${p}_{i+1}={{\beta }_{i}p}_{i}- {\nabla }_{\theta }J\left({\theta }_{i+1}\right),$$

where ${\alpha }_{i}$ is the step size found by line search, ${p}_{i}$ is the search direction and initialized by $- {\nabla }_{\theta }J({\theta }_{0})$ and ${\beta }_{i}$ is a scalar coefficient.

Also, as shown in Eqs. (3) and (7), the factors ${\beta }_{\text{i}}$ and $\beta$ are similar for combining past information gained from past calculation to the current gradient, but $\upbeta$ in Eq. (3) is fixed and ${\beta }_{\text{i}}$ is adaptive in Eq. (7). In contrast, the two methods are different in complexity where momentum just applies fixed momentum and fixed step size; on the other hand, NCG needs a line search procedure to calculate ${\alpha }_{i}$ every iteration, which makes momentum simpler to implement. So, on the same update rules of the momentum method, we use the adaptive momentum factor ${\upbeta }_{\text{i}}$ which has many formulas in the literature like Fletcher–Reeves^28^, Polak–Ribiere–Polyak^30,33^, Hestenes–Stiefel^32^ and Dai–Yuan^34^. Fletcher–Reeves Eq. (8) is the used formula in our work as an adaptive momentum formula.

8 $${\beta }_{\text{i}}^{FR}= \frac{{g}_{i}^{T} . {g}_{i}}{{g}_{i-1}^{T} . {g}_{i-1}}.$$

Unlike Wang and Ye^27^, which focuses solely on momentum adaptation via NCG-inspired updates, DESGD integrates two complementary adaptive mechanisms:

- The Fletcher–Reeves formula for momentum adaptation, and
- A cosine-based step size adjustment derived from recent gradient alignment techniques^35^.

This dual approach allows DESGD to dynamically respond to both the history of gradients (via momentum) and the consistency of descent direction (via step size), without resorting to computationally expensive line searches as seen in Ref.^29^ or Armijo-based methods^36^.

Furthermore, we introduce two novel truncation schemes—clipping and reciprocal—to ensure the adaptive momentum coefficient remains within the stable interval [0,1), a consideration not addressed in Ref. 27.

Besides the application of adaptive momentum, we truncate the value of this factor to meet the condition of momentum method that the momentum factor has to be in the range between $[\text{0,1})$.

If the value of ${\beta }_{i}\ge 1$**,** We have two different schemes:

(1) Clipping

9 $${\beta }_{i}=\text{min}\left({\beta }_{i},\upepsilon \right),$$

where $\epsilon$ is any value close to 1, in our work $\upepsilon =0.99$.

(2) Reciprocal

10 $${\beta }_{i}=1/{\beta }_{i}.$$

By using Eq. (8) we guarantee that ${\beta }_{i}$’s value will not be negative value and the two schemes are valid.

To adapt the step size in Eq. (3), we follow the concept of calculating the cosine of the angle between gradient directions in consecutive iterations. This concept was formulated in Ref. 37 by the following:

11 $$\text{cos}{\varnothing }_{i}=\frac{-{\nabla }_{\uptheta }\text{J}\left({\uptheta }_{i}\right) \cdot \Delta {\uptheta }_{i-1}}{\Vert {\nabla }_{\uptheta }\text{J}\left({\uptheta }_{i}\right)\Vert \Vert \Delta {\uptheta }_{i-1}\Vert }$$

and used by Refs. 38–40 with different ways to update the step size. New formula^35^ was proposed in which is

12 $$\text{cos}{\varnothing }_{i}=\frac{{\nabla }_{\theta }J\left({\theta }_{i}\right) \cdot {\nabla }_{\theta }J\left({\theta }_{i-1}\right)}{\Vert {\nabla }_{\theta }J\left({\theta }_{i}\right)\Vert \Vert {\nabla }_{\theta }J\left({\theta }_{i-1}\right)\Vert }.$$

**Fig. 1:**
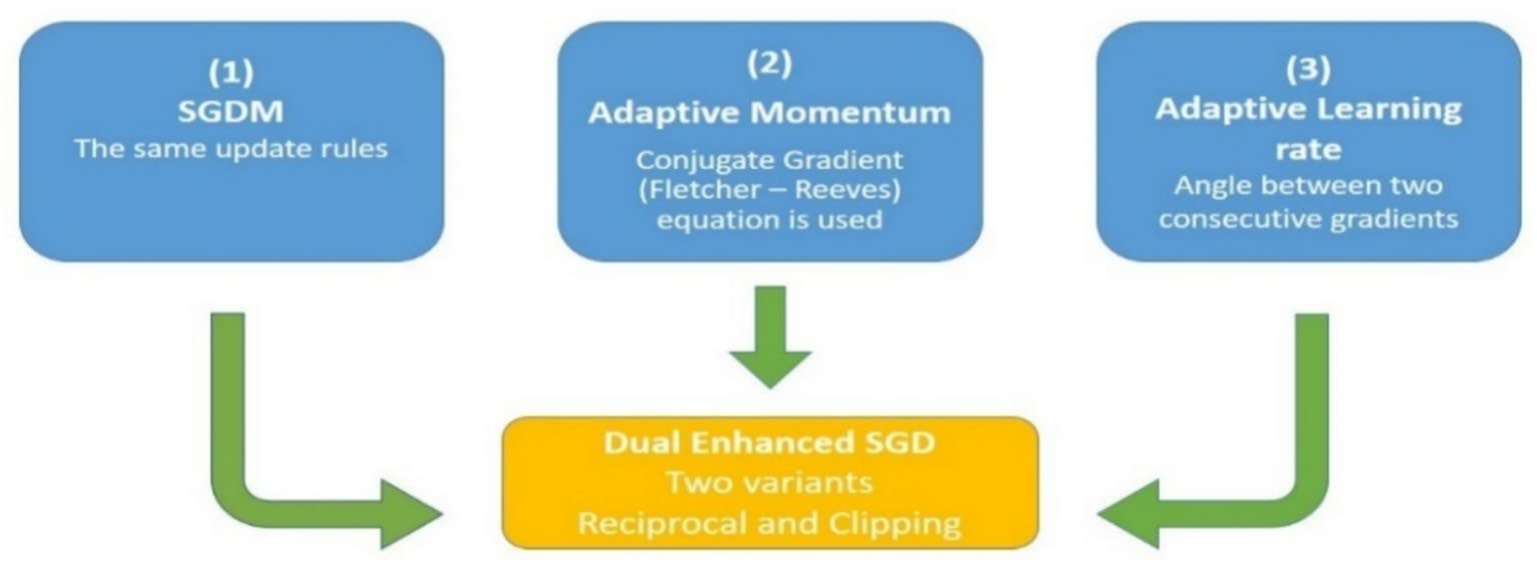
Components of dual enhanced SGD (DESGD) algorithm.

**Fig. 2:**
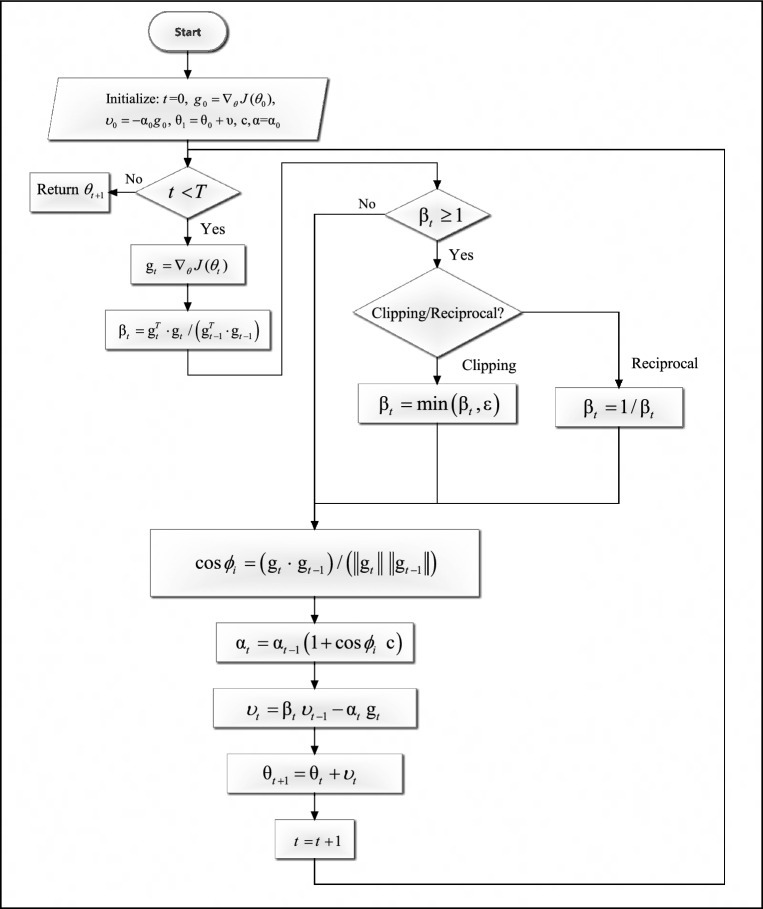
Dual enhanced SGD (DESGD) algorithm procedure.

Equation (12) was applied in two ways which are c2min and p2min. We choose c2min to be combined in our work.

13 $${\alpha }_{i}= {\alpha }_{i-1}\left(1+\text{cos} {\varnothing }_{i}.c\right),$$

where $c =0.5$. We study more factors for constant $c$ which are reciprocal of powers of 2 such as $\frac{1}{2},\frac{1}{4},\frac{1}{8}$, etc. Algorithm 1 presents the steps of DESGD, Fig. 1 demonstrates the previously discussed main components of DESGD and Fig. 2 shows the flowchart of the algorithm. Tables 1 and 2 demonstrate the comparison between the proposed DESGD with Wang & Ye^27^ and other methods, respectively.

**Table 1:** Comparison between Wang and Ye^27^ with the proposed DESGD.

Aspect	Wang & Ye^27^	Proposed DESGD
Adaptation mechanism	Momentum only (Fletcher–Reeves CG-inspired)	Dual adaptation: both momentum (Fletcher–Reeves with truncation) and step size (cosine-based rule)
Momentum stability	No safeguard: ${\beta }_{t}$ may exceed 1, risking divergence	Two novel truncation schemes: (i) Clipping ${\beta }_{t}=\text{min}({\beta }_{t},0.99)$ (ii) Reciprocal $\beta t=\frac{1}{{\beta }_{t}}.$
Step size update	Fixed step size	Adaptive cosine-based update ${\alpha }_{i}= {\alpha }_{i-1}(1+\text{cos} {\varnothing }_{i}.c)$; lightweight, no line search
Computational overhead	Low; The only overhead is the computation of one extra inner product for the FR ratio	Low; avoids expensive line searches, only inner products of gradients for cosine-based adaptive step size and FR ratio
Theoretical analysis	Convergence for momentum on quadratics only	(i) Stability of momentum under truncation (ii) Descent guarantee for step size (iii) Convergence under stochastic convex settings

**Table 2:** Comparison between the proposed DESGD and other methods.

Method	Adaptive momentum	Adaptive step size	Line-search free	Theoretical guarantees
Wang & Ye^27^	✓	×	✓	Yes
Hao et al.^29^	✓	✓(via QLS)	×	Yes
Vaswani et al.^36^	×	✓(Armijo)	×	Yes
DESGD (Ours)	✓	✓(cosine-based)	✓	Yes (this work)

**Algorithm 1:**
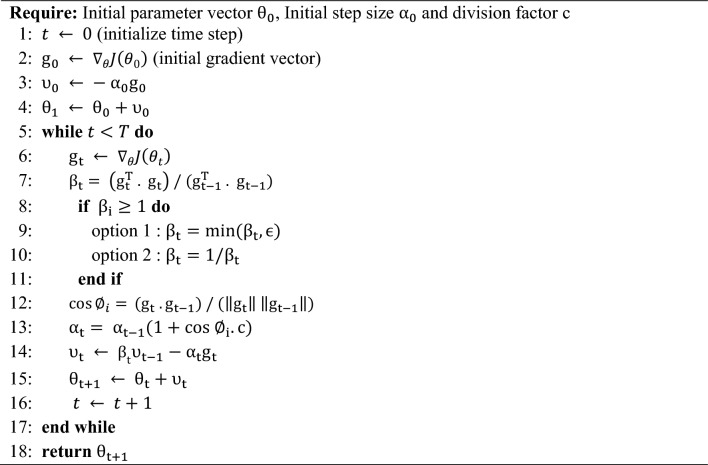
Dual enhanced SGD algorithm (DESGD).

## Theoretical foundations and convergence properties

This section establishes the theoretical guarantees of the proposed dual enhanced stochastic gradient descent (DESGD) method. We analyze the stability of the adaptive momentum update, the descent behavior under cosine-based step-size adaptation, and the overall convergence rate under standard convexity assumptions.

### Preliminaries and assumptions

Let $J(\theta ):{\mathbb{R}}^{d}\to {\mathbb{R}}$ denote a continuously differentiable objective function with gradient ${g}_{t}=\nabla J({\theta }_{t})$. The DESGD update rules are

$${\upupsilon }_{t} = {\beta }_{t} {\upupsilon }_{t-1} - {\alpha }_{t} {g}_{t},\hspace{1em}{\theta }_{t+1} = {\theta }_{t} + {\upupsilon }_{t},$$

where the adaptive coefficients *β*_*t*_ and *α*_*t*_ are computed using Eqs. (8)–(13).

$${\it{A1}}\,\, L$$ *-smoothness*

$J(\theta )$ Has an *L*-Lipschitz continuous gradient, i.e.

$$J(y) \le J(x)+\nabla J{(x)}^{T}(y-x)+(L/2){\| y-x\| }^{2}, \forall x,y.$$

*A2. Bounded gradients*

There exists $G>0$ such that $\| {g}_{t}\| \le G$ for all $t$.

*A3. Stable momentum*

The adaptive momentum factor satisfies ${\beta }_{t} \in [\text{0,1})$, guaranteed by the clipping and reciprocal truncation schemes in Eqs. (9) and (10).

*A4. Unbiased stochastic gradients*

For stochastic optimization, $\text{E}[{g}_{t}|{\mathcal{F}}_{t}] = \nabla J({\theta }_{t})$ and $\text{E}\| {g}_{t}-{\nabla J({\theta }_{t})\| }^{2}\le {\sigma }^{2}$.

### Stability of the velocity update

#### Lemma 1 (velocity boundedness).

Under A2–A3, the velocity sequence defined by ${\upupsilon }_{t}={\beta }_{t} {\upupsilon }_{t-1}-{\alpha }_{t} {g}_{t}$ satisfies

$$\| {\upupsilon }_{t}\| \le {\beta }_{t}\| {\upupsilon }_{t-1}\| + {\alpha }_{t} G.$$

By recursion,

$$\| {\upupsilon }_{t}\| \le \left({\prod }_{i=1}^{t} {\beta }_{i}\right) \| {\upupsilon }_{0}\| + G \sum_{k=0}^{t-1}\left({\prod }_{i=k+1}^{t} {\beta }_{i}\right){\alpha }_{k+1}.$$

Hence, if sup_t_${\beta }_{t} \le \overline{\beta }<1$ and sup_t_ ${\alpha }_{t} \le \overline{\alpha }< \infty$, then $\| {\upupsilon }_{t}\|$ remains uniformly bounded.

#### Proof.

Apply the triangle inequality:

$$\Vert {\upupsilon }_{t}\Vert = \Vert {\beta }_{t}{\upupsilon }_{t-1}- {\alpha }_{t} {g}_{t}\Vert \le {\beta }_{t} \Vert {\upupsilon }_{t-1}\Vert + {\alpha }_{t}\Vert {g}_{t}\Vert \le {\beta }_{t}\Vert {\upupsilon }_{t-1}\Vert + {\alpha }_{t} G.$$

Iterating yields the stated bound. Since ${\beta }_{t}<1$, the geometric product decays, preventing velocity explosion.

### Descent property of the adaptive step size

#### Proposition 1 (one-step descent inequality).

Let $J$ satisfy A1–A3. Then the DESGD update ensures

$$J\left({\theta }_{t+1}\right)\le J\left({\theta }_{t}\right)- {\alpha }_{t} {\Vert {g}_{t}\Vert }^{2} + {\beta }_{t}G\Vert {\upupsilon }_{t-1}\Vert + L{{\beta }_{t}}^{2}{\Vert {\upupsilon }_{t-1}\Vert }^{2} + L {{\alpha }_{t}}^{2}{G}^{2}.$$

Consequently, if ${\alpha }_{t} \le 1/L$ and $\| {\upupsilon }_{t-1}\|$ is bounded, there exists $c\in (\text{0,1})$ such that

$$J({\theta }_{t+1}) \le J({\theta }_{t}) - c {\alpha }_{t}{\| {g}_{t}\| }^{2},$$

i.e. each iteration yields a descent in the objective function.

#### Proof.

By using $L$-smoothness,

$$J({\theta }_{t+1}) \le J({\theta }_{t}) + {g}_{t}^{T}({\theta }_{t+1}-{\theta }_{t}) + (L/2){\| {\theta }_{t+1}-{\theta }_{t}\| }^{2}.$$

Using $${\theta }_{t+1}-{\theta }_{t} = {\upupsilon }_{t}= {\beta }_{t} {\upupsilon }_{t-1} - {\alpha }_{t} {g}_{t}$$ gives

$$J({\theta }_{t+1}) \le J({\theta }_{t}) - {\alpha }_{t}{\| {g}_{t}\| }^{2} + {\beta }_{t} {g}_{\text{t}}^{T} {\upupsilon }_{t-1} + (L/2){\| {\upupsilon }_{t}\| }^{2}.$$

Bound the last two terms: $|{g}_{t}^{T} {\upupsilon }_{t-1}| \le G\| {\upupsilon }_{t-1}\|$ and, by Lemma 1, $\| {\upupsilon }_{t}\| \le {\beta }_{t}\| {\upupsilon }_{t-1}\| + {\alpha }_{t}G$.

Hence, ${\| {\upupsilon }_{t}\| }^{2} \le {2{\beta }_{t}}^{2}{\| {\upupsilon }_{t-1}\| }^{2} + 2{{\alpha }_{t}}^{2}{G}^{2}.$ Substituting yields the inequality. When ${\alpha }_{t} \le 1/L$ and $\| {\upupsilon }_{t-1}\|$ is bounded, the negative term $-{\alpha }_{t} {\| {g}_{t}\| }^{2}$ dominates, ensuring descent.

### Convergence under stochastic convex setting

#### Theorem 1 (Convergence and regret bound).

Assume $J(\theta )$ is *μ*-strongly convex with $L$-Lipschitz gradients and that A2–A4 hold.

If ${\alpha }_{t} = {\alpha }_{0}/(1+\gamma t)$ for some ${\alpha }_{0} \le 1/L$ and $\gamma > 0$, and ${\beta }_{t} \in [\text{0,1})$, then DESGD satisfies

$$\text{E}[{R}_{T}] = \sum_{t=1}^{T}\text{E}[J({\theta }_{t})-{J}^{*}] = O(1)$$

and

$$\text{E}\left[J\left({\overline{\theta }}_{T}\right)-{J}^{*}\right]= O\left(\frac{1}{T}\right),$$

where ${\overline{\theta }}_{T}= (1/T) \sum_{t=1}^{T}{\theta }_{t}$ denotes the averaged iterate.

#### Proof.

Taking conditional expectation of (A4) and using unbiased gradients,

$$\text{E}[J({\theta }_{t+1}) | {\mathcal{F}}_{t}]\le J({\theta }_{t} )-{\alpha }_{t} {\| \nabla J({\theta }_{t})\| }^{2}+{\beta }_{t} G\text{ E}[{\| \nabla J({\theta }_{t})\| }^{2}\mid {\mathcal{F}}_{t}]+L\text{ E}[{\| {\upupsilon }_{t}\| }^{2}].$$

Under strong convexity,

$${\Vert \nabla J\left({\theta }_{t}\right)\Vert }^{2}\ge 2\mu \left[J\left({\theta }_{t}\right)-{J}^{*}\right].$$

Substituting this bound gives

$$\text{E}[J({\theta }_{t+1})-{J}^{*}] \le (1-2\mu {\alpha }_{t})\text{E}[J({\theta }_{t})-{J}^{*}] + {C}_{1} {{\alpha }_{t}}^{2} + {C}_{2} {{\beta }_{t}}^{2}$$

for constants ${C}_{1},{C}_{2}$ depending on $L,G$. Applying this inequality recursively and using summing over $\text{t}$ and using $\sum {\alpha }_{t}= \infty , \sum {{\alpha }_{t}}^{2} < \infty$, we obtain the sublinear convergence rate

$${\rm E}\left[J\left({\overline{\theta }}_{T}\right)- {J}^{*}\right] = O \left(\frac{1}{T}\right).$$

For constant ${\alpha }_{t} \le 1/L$, the iterates converge linearly to a neighborhood of size $O(\alpha {\sigma }^{2}).$

## Experiments and results

In this section we introduce the experiments done to compare our method and other methods like SGD with momentum (SGDM)^9^ and Adam^14^. In Numerical tests section , we tested three methods on a set of unconstrained optimization test functions, and in Neural network section , we designed a simple neural network experiment to demonstrate the efficiency of our method on a machine learning task. All the experiments were done with the Python programming language and the Numpy package. Intel(R) Core(TM) i3-1005G1 CPU @ 1.20GHz was used with 12GB RAM.

### Numerical tests

**Fig. 3:**
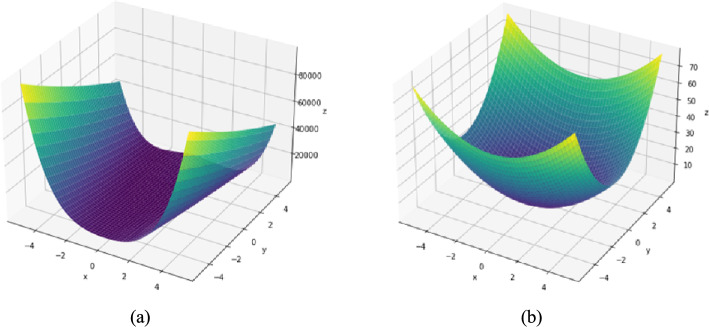
(**a**) Rosenbrock test function. (**b**) Sum square test function.

Two optimization test functions from Refs. 41 and 42 are investigated to test our proposed algorithm numerically, which are Rosenbrock function and Sum Square function, shown in Fig. 3. The three methods, DESGD, SGDM and Adam, are compared according to the number of iterations and CPU time. The stopping criteria used is $\| {g}_{k} \| \le {10}^{-6}$ as suggested by K. E. Hillstrom in Ref. 43. To get the best results for all methods in this task, the step sizes are tuned from 0.9 descending to ${10}^{-9}$ and substitute with each value once at a time, the best value of step size got the best number of iterations for each method. For SGDM, the method is tested as a gradient descent method with the same update rules and with different fixed values for momentum.

parameter like 0.9, 0.99, 0.8 and 0.5. The same values of momentum parameter are applied to tune ${\beta }_{1}$ and ${\beta }_{2}$ in Adam. The results of Rosenbrock and Sum Square functions and the initial points used in this study are displayed in Tables 3 and 4, respectively.

**Table 3:** The results for 2D-Rosenbrock function.

	NOI	CPU time	Error
(3,3)			
SGDM	3448	62.8696	3.507e–07
Adam	2406	60.6331	2.023e–06
FR_clip	660	21.4483	2.432e–06
FR_rec	801	32.1379	2.428e–06
(20,20)			
SGDM	80,747	1471.4568	2.503e–06
Adam	13,756	359.8340	1.230e–06
FR_clip	4267	148.3628	2.419e–06
FR_rec	4006	130.7945	2.474e–06
(100,100)			
SGDM	> ${10}^{5}$	**–**	124.329
Adam	39,474	1202.1233	1.398e–06
FR_clip	25,334	798.5497	2.400e–06
FR_rec	39,633	1366.9392	2.454e–06

**Table 4:** The results for 2D-sum square function.

	(4,4)			(25,25)		
	NOI	CPU time	Error	NOI	CPU time	Error
SGDM	46	1.1956	5.124e–07	51	1.0449	8.825e–07
Adam	45	1.3682	1.605e–06	76	2.0103	1.219e–05
FR_clip	15	0.5613	7.221e–08	17	0.6368	9.243e–08
FR_rec	15	0.5289	7.221e–08	17	0.6039	9.243e–08

### Neural networks

**Fig. 4:**
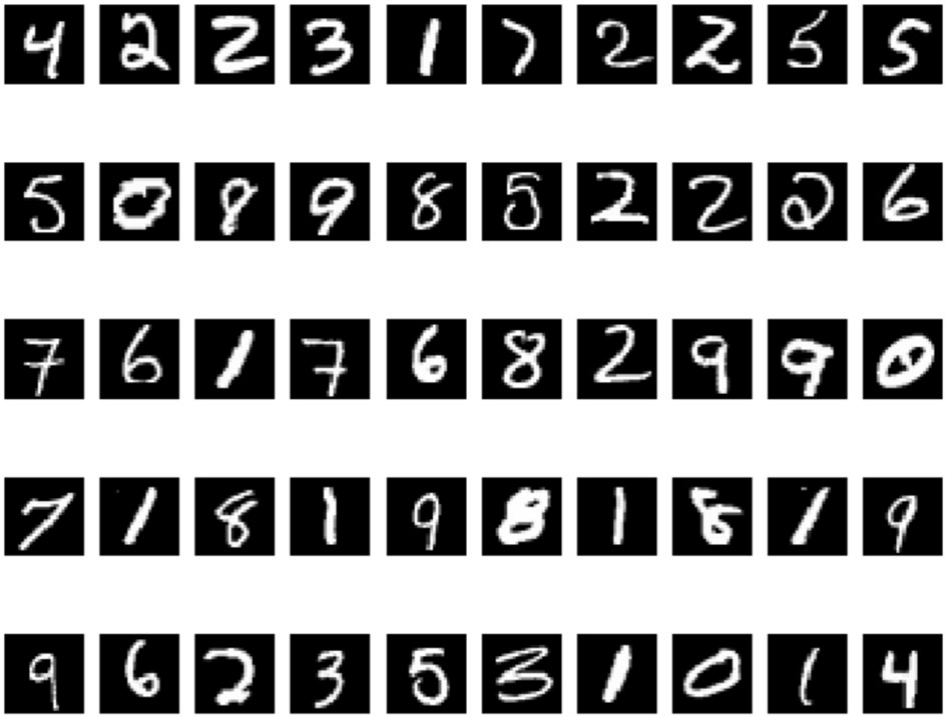
Samples from handwritten numbers (MNIST) dataset.

The MNIST dataset is used, a sample is shown in Fig. 4, to identify handwritten single digits in $28\times 28$ pixel images. A simple multilayer perceptron (MLP) with 2 hidden layers (256 neuron in each layer) is designed to obtain the results of the three methods with three batch sizes: 256, 128 and 64. The results shown in Table 5 state that the SGD with adaptive momentum and adaptive step size can perform better than SGD with fixed momentum and slightly better than Adam on such neural networks. The hyperparameters for SGDM and Adam were set to their default values, which yielded the best performance in our experiments: a learning rate of 0.01 and momentum of 0.9 for SGDM, and a learning rate of 0.001, *β*₁ = 0.9, and *β*₂ = 0.999 for Adam.

**Table 5:** The test results for MLP on MNIST dataset.

	Batch size = 256		Batch size = 128		Batch size = 64	
	Accuracy	loss	Accuracy	loss	Accuracy	loss
SGDM	94.99	0.0329	96.13	0.02369	97.27	0.0169
Adam	96.97	0.0199	97.13	0.01790	97.45	0.0233
FR_clip	96.43	0.0221	97.38	0.01512	97.91	0.0143
FR_rec	95.33	0.0290	97.06	0.01789	97.88	0.0144

*NOI*: Number of iterations the optimizer has accomplished.

*CPU* *Time*: The total CPU time needed by the optimizer to accomplish NOI and it measured in msec.

*Error*: The distance from the final point that reached by the optimizer and the minimum point and is measured using $\Vert x-{x}^{*}\Vert$ where $x$ is the final point and ${x}^{*}$ is the minimum point.

*SGDM*: SGD with momentum (fixed momentum).

*FR_clip*: Fletcher-Reeves adaptive momentum truncated by clipping.

*FR_rec*: Fletcher-Reeves adaptive momentum truncated by the reciprocal.

## Discussion

**Fig. 5:**
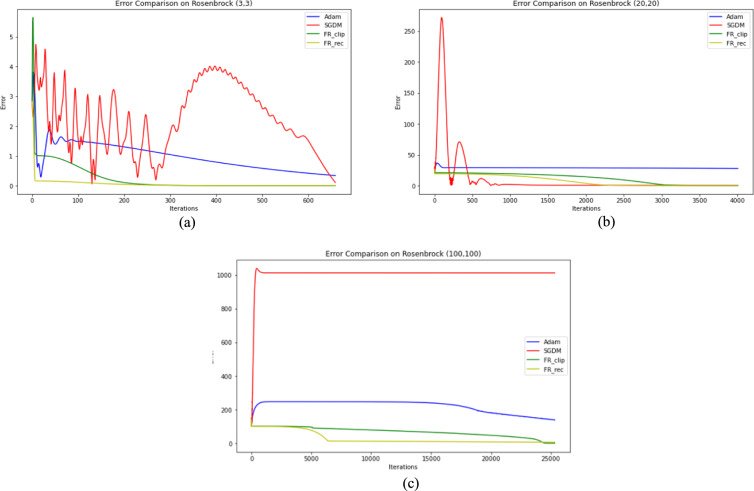
Error comparison between different optimization methods for different starting points on Rosenbrock function (**a**) Point (3, 3)**,** (**b**) Point (20, 20), (**c**) point (100, 100).

**Fig. 6:**
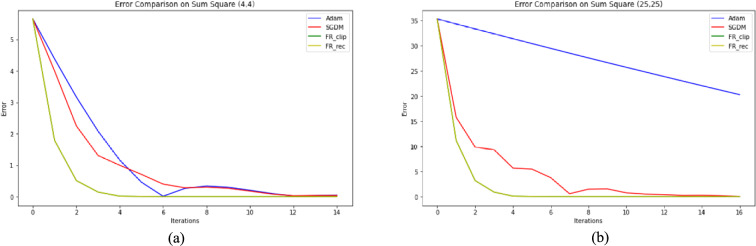
Error comparison between different optimization methods for different starting points on Sum Square function. (**a**) Point (4, 4), (**b**) Point (25, 25).

The results obtained in Tables 3 and 4 show that the DESGD with its two versions performs better in terms of iterations and time across all starting points as shown in Figs. 5 and 6, even when starting far away. It’s more robust than standard SGDM, especially in high starting points like (100, 100) where SGDM fails or due to the oscillations that slow down the convergence. Adam is reliable but takes more iterations and time compared to the DESGD. The error DESGD is slightly higher than Adam in some cases but still meets the required threshold.

Another aspect to be considered is computational efficiency. The time per iteration for Adam ranges from 0.025 to 0.030 ms/iteration while for DESGD ranges from 0.031 to 0.035 ms/iteration. So DESGD may have a higher per-iteration cost but still ends up being faster overall because it needs way fewer steps. That suggests the modification might be adding some computation per step, but the reduction in iterations compensates.

**Fig. 7:**
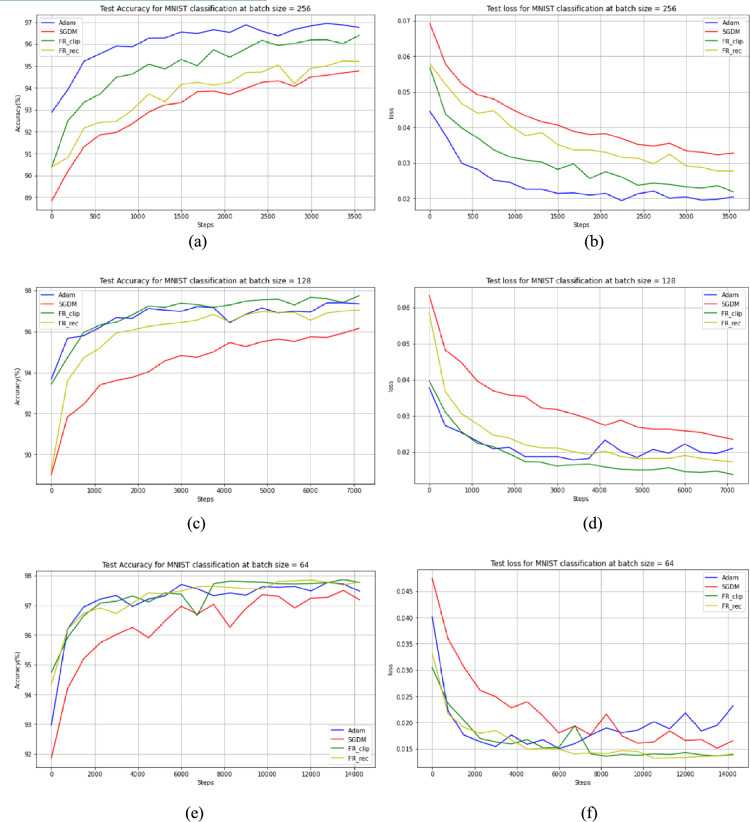
Test Accuracy and loss for different batch sizes on MNIST dataset. (**a**) Test Accuracy for batch size = 256, (**b**) Test loss for batch size = 256, (**c**) Test Accuracy for batch size = 128, (**d**) Test loss for batch size = 128, (**e**) Test Accuracy for batch size = 64 and (**f**) Test loss for batch size = 64.

On the other hand, the neural network experiment shows that the modifications done on SGDM with adaptive momentum and adaptive step size can boost the performance of this optimizer. The best experimental value for constant c is $1/1024$ which shows the best results for different batch sizes. DESGD has been evaluated against the best tuned SGDM and Adam at various batch sizes as seen in Table 5. The results obtained from Fig. 7 have been calculated from multiple runs. Also, the results show that FR_clip version of DESGD provide the best performance of the two versions. Practically, the best step size to train such neural networks is 0.1 for DESGD which is considered a consequence of the modifications that has been done on the original SGDM.

As shown in Table 6, despite consistently achieving the lowest per-step processing time (6.75–9.06 ms) across all batch sizes, SGDM’s efficiency is at the expense of its predictive performance. Adam’s adaptive learning method keeps it popular even if it takes 40–80% longer than SGDM (12.94–16.39 ms). In comparison to Adam (15.37–18.15 ms), our suggested approach, DESGD with its two variants, adds an additional 10–30% overhead, or about double the price of SGDM. Its higher classification accuracy and robustness on MNIST, however, outweigh this added computing burden. Crucially, the relative order of methods does not change with batch size, showing that the cost of DESGD increases proportionately with data size instead of skyrocketing with smaller batches. Together, these results show that even though the suggested approaches need somewhat more computation per step, they are still competitive with Adam and SGDM because they provide a better balance between efficiency and predictive performance, which is especially useful in applications where accuracy is more important than speed.

**Table 6:** Computational time per step in milliseconds (ms) for MLP on MNIST dataset.

	Batch size = 256	Batch size = 128	Batch size = 64
SGDM	9.06	7.45	6.75
Adam	16.39	12.94	14.22
FR_clip	18.15	16.90	15.83
FR_rec	17.69	16.73	15.37

Another point is the computational cost of some proposed methods regarding adaptive step size that were tested on stochastic gradient descent or combined with the conjugate gradient to adapt the momentum parameter as we did. Hao^29^ used a quadratic line search method with two schemes, two-point interpolation and least square estimation to adapt step size besides using conjugate gradient formula (like Polak–Ribiere) and this method need more function and gradient evaluations in each scheme of the quadratic line search. Vaswani^36^ combined stochastic gradient descent with a stochastic variant of the classic Armijo line search and this method has to hold the classic Armijo conditions which require function and gradient evaluations for each mini batch. In contrast, we adapt the step size depending on the angle between the two vectors, the gradient and the previous gradient without the need to do auxiliary function or gradient evaluations.

## Conclusion

In this work, a novel optimization technique, dual enhanced stochastic gradient descent (DESGD), that combines the strengths of stochastic gradient descent with momentum (SGDM) and conjugate gradient descent (CG) components is proposed. By adjusting the momentum factor in SGDM using Fletcher–Reeves equation from CG while maintaining the momentum term within [0, 1) and incorporating an adaptive step size method, the approach enhances stability and convergence. The mathematical foundation of the method introduced the convergence analysis and validated it through several experiments, where the optimizer was applied to a neural network model trained on the benchmark dataset MNIST and examined on well-known optimization test functions like Rosenbrock and Sum square functions. Performance was assessed against widely used optimizers using standard metrics, which demonstrated the effectiveness of the proposed technique.

While our initial tests were limited to a few simple models and datasets, the results are really encouraging and point to several exciting paths we want to explore next. We also only tested a handful of optimizers, and some settings still need hand-tuning.

Building on this solid start, our next steps are to make the optimizer smarter and more automatic. Ways for it to dynamically adjust its own momentum and step size will be developed, and a method to automatically choose key parameters will be created. To really prove its strength, it will be tested on more complex designs—like CNNs, RNNs, and Transformers trained on large datasets. Finally, it will be put to work on real-world problems to truly demonstrate how scalable and useful it can be.

## Data Availability

No publicly available repositories or databases are suitable for the current data submission. All data supporting the results of this study are available in the article. They can also be obtained from the corresponding author, MF, upon reasonable request.
